# Genome sequence of the sulfur-oxidizing *Bathymodiolus thermophilus* gill endosymbiont

**DOI:** 10.1186/s40793-017-0266-y

**Published:** 2017-09-02

**Authors:** Ruby Ponnudurai, Lizbeth Sayavedra, Manuel Kleiner, Stefan E. Heiden, Andrea Thürmer, Horst Felbeck, Rabea Schlüter, Stefan M. Sievert, Rolf Daniel, Thomas Schweder, Stephanie Markert

**Affiliations:** 1 Institute of Pharmacy, Ernst Moritz Arndt University, Greifswald, Germany; 2 Max Planck Institute for Marine Microbiology, Department of Symbiosis, Bremen, Germany; 3 Department of Geoscience, University of Calgary, Calgary, Canada; 4 Department of Genomic and Applied Microbiology & Göttingen Genomics Laboratory, Georg August University, Göttingen, Germany; 5 Scripps Institution of Oceanography, La Jolla, CA USA; 6 Imaging Center of the Department of Biology, Ernst Moritz Arndt University, Greifswald, Germany; 7 Woods Hole Oceanographic Institution, Biology Department, Woods Hole, MA USA; 8 Institute of Marine Biotechnology, Walther-Rathenau-Straße 49A, 17489 Greifswald, Germany

**Keywords:** Uncultured endosymbiont, Hydrothermal vents, Marine invertebrate symbiosis, Thiotrophy, Autotrophy

## Abstract

*Bathymodiolus thermophilus*, a mytilid mussel inhabiting the deep-sea hydrothermal vents of the East Pacific Rise, lives in symbiosis with chemosynthetic *Gammaproteobacteria* within its gills. The intracellular symbiont population synthesizes nutrients for the bivalve host using the reduced sulfur compounds emanating from the vents as energy source. As the symbiont is uncultured, comprehensive and detailed insights into its metabolism and its interactions with the host can only be obtained from culture-independent approaches such as genomics and proteomics. In this study, we report the first draft genome sequence of the sulfur-oxidizing symbiont of *B. thermophilus*, here tentatively named *Candidatus* Thioglobus thermophilus. The draft genome (3.1 Mb) harbors 3045 protein-coding genes. It revealed pathways for the use of sulfide and thiosulfate as energy sources and encodes the Calvin-Benson-Bassham cycle for CO_2_ fixation. Enzymes required for the synthesis of the tricarboxylic acid cycle intermediates oxaloacetate and succinate were absent, suggesting that these intermediates may be substituted by metabolites from external sources. We also detected a repertoire of genes associated with cell surface adhesion, bacteriotoxicity and phage immunity, which may perform symbiosis-specific roles in the *B. thermophilus* symbiosis.

## Introduction

Chemoautotrophic bacteria form the base of the food-chain in deep sea hydrothermal vent ecosystems [1, 2]. Many of these chemoautotrophs live in highly integrated symbiotic associations with invertebrate hosts, such as mussels, clams and tube worms, which enables megafaunal communities to thrive in the otherwise uninhabitable vent ecosystem [3–5].

The mussel *Bathymodiolus thermophilus*, for example, a bivalve belonging to the family *Mytilidae*, densely populates the hydrothermal vent fields of the Galapagos Rift and of the East Pacific Rise between the latitudes 13 °N and 21 °S [6]. Although the animal’s food groove and digestive tract are reduced [6], *B. thermophilus* appears to be able to ingest and assimilate suspended particles by filter feeding [7].

The major part of the bivalve’s nutrition, however, is derived from its chemosynthetic symbionts [4, 8]. The sulfur-oxidizing bacteria live within specialized gill cells, so called bacteriocytes [9]. Provided with a steady supply of reduced sulfur from the vents, these symbionts synthesize organic compounds and thus feed their host [10, 11].

Investigations on the symbiont’s physiology have hitherto been limited by the inaccessibility of mussel samples and failure to culture the symbionts in vitro. Underlying metabolic pathways that facilitate the putative inter-exchange of nutrients between the symbiotic partners therefore remain unexplored. However, culture-independent methods, such as direct genomic, transcriptomic or proteomic analyses of symbiont-containing tissue or of enriched symbiont fractions have provided useful physiological information about various uncultured marine symbionts in the past [12–17]. In this study we used symbiont-enriched preparations from *B. thermophilus* gill tissue to assemble the first draft genome of the *B. thermophilus* symbiont in order to gain preliminary insights into its metabolic potential.

## Organism information

### Classification and features

**Fig. 1:**
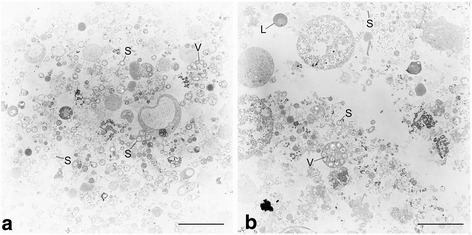
Transmission electron micrographs of *Candidatus* Thioglobus thermophilus. *B. thermophilus* gill tissue was homogenized in a glass tissue grinder and subjected to crude density gradient centrifugation using Histodenz™ gradient medium. Subsamples were taken from two visible bands and fixed for electron microscopy (**a** and **b**). Both subsamples contained numerous free symbiont cells (S) as well as some intact host vacuoles (V) containing several symbiont cells, besides various other cellular components and host tissue debris. L: Lipid drop or mucus. Scale bar: 5 μm. Electron microscopy method details: samples were fixed in **a**) 1% glutaraldehyde, 2% paraformaldehyde in IBS (imidazole-buffered saline; 0.49 M NaCl, 30 mM MgSO_4_*7H_2_O, 11 mM CaCl_2_*2H_2_O, 3 mM KCl, 50 mM imidazole) and **b**) in 2.5% glutaraldehyde, 1.25% paraformaldehyde in IBS. After embedding in low-gelling agarose and postfixation in 1% osmium tetroxide in cacodylate buffer (0.1 M cacodylate; pH 7.0), samples were dehydrated in a graded ethanol series (30 to 100%) and embedded in a mixture of Epon and Spurr (1:2). Sections were cut on an ultramicrotome (Reichert Ultracut, Leica UK Ltd., Milton Keynes, UK), stained with 4% aqueous uranyl acetate for 5 min followed by lead citrate for 1 min and analyzed with a transmission electron microscope LEO 906 (Zeiss, Oberkochen, Germany)

*B. thermophilus* symbiont cells are coccoid or rod-shaped (Fig. 1). In electron micrographs, they typically appear as roundish forms, whose central region is light or transparent (looking “empty”), while the outermost regions of the cytoplasm are darker and more structured (Fig. 1 and [9]). Like most sulfur-oxidizing (thiotrophic) bivalve symbionts [4, 18], the *B. thermophilus* symbiont has a Gram-negative cell wall. With a diameter of 0.3–0.5 μm, *B. thermophilus* symbiont cells are of similar size as thiotrophic symbionts from other *Bathymodiolus* host species [19–22], and notably smaller than sulfur-oxidizing symbionts from other invertebrate hosts [4, 23]. In the host tissue, the symbionts are usually enveloped in large vacuoles. Groups of up to 20 symbionts within a single host vacuole have previously been reported by Fisher and colleagues [9]. Imaging of purified symbiont fractions from homogenized *B. thermophilus* gill tissue revealed, besides a large number of free symbiont cells, some intact vacuoles encompassing multiple symbionts (Fig. 1).

*B. thermophilus* symbionts reside intracellularly in bacteriocytes in their host’s gill tissue. Unlike some other *Bathymodiolus* species, such as *B. azoricus* that maintains a dual symbiosis with both sulfur-oxidizing and methane-oxidizing bacteria [24], *B. thermophilus* hosts only one type of bacterial endosymbionts. Based on 16S rRNA gene similarity [25], this sulfur-oxidizing symbiont population in *B. thermophilus* belongs to a single phylotype.

**Fig. 2:**
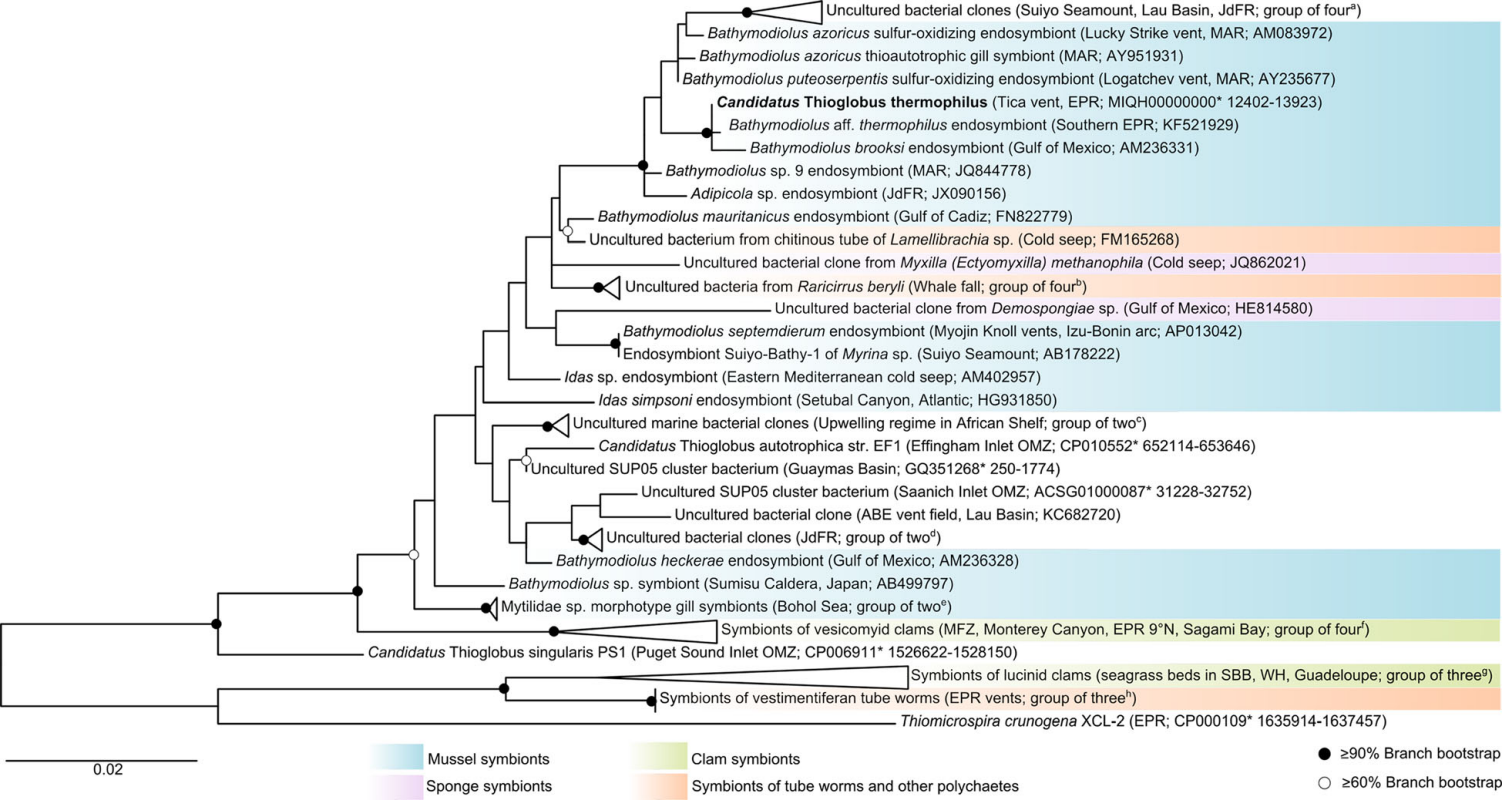
Phylogenetic tree of *Candidatus* Thioglobus thermophilus and related free-living and host-associated sulfur oxidizers. *Ca.* T. thermophilus, the thiotrophic symbiont of *Bathymodiolus thermophilus*, is displayed in bold. The tree was inferred from closely related 16S rRNA gene sequences obtained from the SILVA database using the SILVA Incremental Aligner (SINA) [51] and was estimated with the 16S rRNA sequence of 46 bacteria. The final alignment covered 1138 nucleotides. Sequence alignment and phylogenetic analysis were performed using the MEGA7 software tool [52]. The phylogenetic tree was constructed using the Maximum Likelihood method based on the Tamura-Nei model implemented in MEGA7 [53]. Branch bootstrap support values were calculated using 1000 replicates and are displayed as circles (black: ≥ 90%, white: ≥ 60%). For the sake of clarity some organisms were merged into groups (wedges): ^a^uncultured clones (KC682721, KC682765, JQ678401, AB193934); ^b^whale fall symbionts (HE814589, HE814588, HE814591 HE814585); ^c^uncultured clones (FM246509, FM246513); ^d^uncultured clones (JQ678344, JQ678392); ^e^Mytilidae symbionts (AM503921, AM503923); ^f^Vesicomyidae symbionts (EU403432, EU403431, CP000488* 1081274–1,082,807, AP009247* 948400–949,934); ^g^Lucinidae symbionts (X84979, M99448, M90415); ^h^tube worm symbionts (NZ_AFOC01000137* 503–2033, DQ660821, NZ_AFZB01000059* 4132–5662). The lucinid clam symbionts, the vestimentiferan tube worm symbionts, and the free-living Thiomicrospira crunogena XCL-2 were included as outgroup. Branches that are not highlighted by colors represent free-living relatives. The tree is drawn to scale, with branch lengths measured in the number of substitutions per site. *these NCBI accession numbers refer to whole genome submissions and not to individually submitted 16S rRNA gene sequences (start and stop positions of the 16S rRNA gene are given after the asterisk). JdFR: Juan de Fuca Ridge, EPR: East Pacific Rise, MAR: Mid-Atlantic Ridge, OMZ: oxygen minimum zone, MFZ: Mendocino Fracture Zone, SBB: Santa Barbara Basin, WH: Woods Hole

The *B. thermophilus* symbiont is a member of the 10.1601/nm.2068 (NCBI taxonomy ID 2360). It is closely related to symbionts of other *Bathymodiolus* species, and more distantly related to symbionts of other invertebrate hosts and to free-living 10.1601/nm.2068 from various marine habitats [26]. The *B. thermophilus* symbiont falls in a well-supported clade consisting of symbionts of other mytilid and vesicomyid bivalves and free-living gammaproteobacterial clones from marine vents and other submarine volcanic sites as shown in Fig. 2. Its closest relatives are the ‘*Bathymodiolus* aff. *Thermophilus* thioautotrophic gill symbiont’ from 32 °N EPR (NCBI taxonomy ID 363574; 99.85% similarity on the 16S rRNA level) and the *Bathymodiolus brooksi* symbiont from the Gulf of Mexico (NCBI taxonomy ID 377144; 99.53% similarity). According to our analysis, the *B. thermophilus* symbiont is only remotely similar to the sulfur-oxidizing symbionts of deep-sea vestimentiferan tube worms (90% 16S rRNA similarity) and of shallow water lucinid clams (87–90% similarity, see Fig. 2).

The *B. thermophilus* symbiont’s closest cultured relative is the free-living *Candidatus* Thioglobus autotrophica, whose genome was recently sequenced [27]. The metabolic properties of both bacteria appear to be highly similar, as predicted from their genomes. Like the *B. thermophilus* symbiont genome presented in this study, the *Ca.* T. autotrophica genome encodes an incomplete TCA cycle. The high degree of 16S rRNA gene sequence similarity between *Ca.* T. autotrophica and the *B. thermophilus* symbiont (95%), suggests that both belong to the same genus. We therefore propose the tentative name *Candidatus* Thioglobus thermophilus for the thiotrophic *B. thermophilus* symbiont.

A summary of key features of *Ca.* T. thermophilus is given in Table 1.

**Table 1:** Classification and general features of *Candidatus* Thioglobus thermophilus, the *Bathymodiolus thermophilus* gill endosymbiont, according to the MIGS recommendations [42]

MIGS ID	Property	Term	Evidence code ^a^
	Classification	Domain Bacteria	TAS [43]
		Phylum *Proteobacteria*	TAS [44]
		Class *Gammaproteobacteria*	TAS [45]
		Order Unclassified	NAS
		Family Unclassified, S-oxidizing symbionts	TAS [46]
		Genus *Candidatus* Thioglobus	TAS [25]
		Species *Candidatus* Thioglobus thermophilus	TAS [25]
		(Type) strain NA	NAS
	Gram stain	Negative	TAS [18]
	Cell shape	Coccoid or short rods	TAS [9], IDA
	Motility	Non-motile inside the host; a putative free-living stage of the symbiont is likely motile, but no direct evidence exists	NAS
	Sporulation	Unknown	
	Temperature range	4 °C - 14 °C (shows psychrophilic growth characteristics)	TAS [10]
	Optimum temperature	Unknown	
	pH range; Optimum	6.9–8.0; unknown	TAS [47]
	Carbon source	CO_2_ (autotroph)	TAS [10, 11, 48, 49]
	Energy source	H_2_S and S_2_O_3_^2−^ (chemotroph)	TAS [10, 49]
	Terminal electron acceptor	O_2_, NO_3_^2−^	TAS [11]
MIGS-6	Habitat	Intracellular endosymbiont of marine bivalve inhabiting hydrothermal vents	IDA
MIGS-6.3	Salinity	35.42 PSU	IDA
MIGS − 22	Oxygen	Aerobic (facultative)	TAS [11]
MIGS-15	Biotic relationship	Symbiont	TAS [10, 11]
MIGS-14	Pathogenicity	Non-pathogenic	NAS
MIGS-4	Geographic location	East Pacific Rise (EPR) 9°N	IDA
MIGS-5	Sample collection	January 2014	IDA
MIGS-4.1	Latitude	9° 50.39′ N	IDA
MIGS-4.2	Longitude	104° 17.49′ W	IDA
MIGS-4.3	Altitude	-2511 m	IDA

^a^Evidence codes - *IDA* Inferred from Direct Assay, *TAS* Traceable Author Statement (i.e., a direct report exists in the literature), *NAS* Non-traceable Author Statement (i.e., not directly observed for the living, isolated sample, but based on a generally accepted property for the species, or anecdotal evidence). These evidence codes are from the Gene Ontology project [50]

## Genome sequencing information

### Genome project history

The genome of *Candidatus* Thioglobus thermophilus was sequenced to get a comprehensive insight into the metabolic potential of the bacterium. This project is part of a larger effort to compare the symbiont genomes from various *Bathymodiolus* species across different vent habitats in order to understand the possible effects of vent geochemistry in shaping host-symbiont evolution in *Bathymodiolus*. Sequencing and assembly of the symbiont genome were conducted at the Göttingen Genomics Laboratory (University of Göttingen, Germany) and at the Max-Planck-Institute of Marine Microbiology (Bremen, Germany), respectively. The sequences have been deposited in GenBank under the accession number MIQH00000000. A summary of the project information is shown in Table 2.

**Table 2:** Project information

MIGS ID	Property	Term
MIGS-31	Finishing quality	Draft genome
MIGS-28	Libraries used	Illumina 112 bp paired-end library (Nextera)
MIGS-29	Sequencing platforms	Genome analyzer II x
MIGS-31.2	Fold coverage	86×
MIGS-30	Assemblers	SPAdes v. 3.1.1
MIGS-32	Gene calling method	GeneMarkS+ (NCBI PGAP)
	Locus Tag	BGC33
	GenBank ID	o MIQH00000000
	GenBank date of release	11/16/2016
	GOLD ID	-
	BioProject	PRJNA339702
MIGS-13	Source material identifier	-
	Project relevance	Vent ecosystems, Chemosynthetic symbioses, Environmental microbiology

### Growth conditions and genomic DNA preparation

Symbionts for genome sequencing were isolated from one single *B. thermophilus* host individual, which was collected during the R/V *Atlantis* cruise AT26–10 in January 2014. The mussel was collected from a diffuse-flow vent at the Tica vent field on the East Pacific Rise at 9° 50.39′ N, 104° 17.49′ W by the remotely operated vehicle (ROV) *Jason*. After recovery, the animal was dissected on board the research vessel and gill tissue was removed and homogenized in 1× PBS buffer (Dulbecco’s Phosphate Buffered Saline, Sigma-Aldrich order no. D5773). The resulting homogenate was diluted with 1× PBS (ratio 1:3) and subjected to multiple centrifugation steps (differential pelleting): In a first centrifugation step (500 × *g*, 5 min, 4 °C in a tabletop centrifuge using a swing-out rotor), crude host tissue debris and host cell nuclei were removed from the homogenate. The supernatant was centrifuged again (step 2) as described above to pellet residual host nuclei. The new supernatant was now centrifuged at maximum speed (step 3), i.e. at 15,000 × *g* for 20 min at 4 °C using a fixed-angle rotor. The resulting pellet contained enriched bacterial cells and was immediately frozen at −80 °C until genomic DNA preparation.

Genomic DNA was isolated from the purified bacteria using the MasterPure DNA Purification Kit (Epicentre) as recommended by the manufacturer.

### Genome sequencing and assembly

Sequencing of the *B. thermophilus* symbiont genome was performed at the Göttingen Genomics Laboratory using the Illumina Genome Analyzer II x. A Nextera shotgun library was generated for a 112 bp paired-end sequencing run. Sequencing resulted in 7,569,934 paired-end reads. Adaptors were removed from the reads, quality-trimmed (Q = 2) with BBDuk and error-corrected with BBnorm (V35, sourceforge.net/projects/bbmap). The resulting reads were assembled with IDBA-UD [28]. To bin the symbiont genome from the metagenome assembly, we used gbtools [29] based on GC content and sequencing coverage. The corrected reads were mapped against the symbiont genome bin with BBmap and reassembled with SPAdes v. 3.1.1 [30]. This assembly resulted in 1341 contigs longer than 200 bp (1281 scaffolds). The completeness and contamination of the genome was estimated with CheckM [31]. The CheckM test showed 96.98% completeness of the genome with 11.32% contamination and 81.40% strain heterogeneity.

### Genome annotation

All scaffolds were annotated using NCBI’s prokaryotic genome annotation pipeline (https://www.ncbi.nlm.nih.gov/genome/annotation_prok/), which uses the gene caller GeneMarkS+ together with a similarity-based gene detection approach [32, 33]. Predicted proteins were assigned Clusters of Orthologous Groups numbers and Protein Families domains by querying their sequences against the COG Database and the Pfam database, respectively, at NCBI (ftp://ftp.ncbi.nih.gov/pub/mmdb/cdd/little_endian/). Querying was done using the rpsblast application of the BLAST + −2.4.0 package with an E-value cutoff of 1 × 10^−5^ and 1 × 10^−4^, respectively, for COG and Pfam. To manually assign COG categories to the COG numbers returned by rpsblast, the COG category database was downloaded from the COG FTP server (ftp://ftp.ncbi.nih.gov/pub/COG/COG2014/data). For prediction of signal peptides the SignalP 4.1 Server [34], PECAS [35] and Phobius [36] were used. Transmembrane helices and CRISPR loci (CRISPR arrays) were predicted with TMHMM Server v. 2.0 [37] and the CRISPRFinder tool [38], respectively.

## Genome properties

The properties of this genome are summarized in Table 3. The draft genome of the sulfur-oxidizing *B. thermophilus* symbiont contained 3,088,407 bp in 1281 scaffolds >200 bp. The average GC content was 37.7%. A total of 3097 genes were predicted, of which 3045 (98.3%) are predicted protein-encoding genes. The remaining 1.5% and 0.2%, respectively, consisted of RNA genes and pseudo genes. Of the protein-encoding genes, 54.5% and 65.2% were affiliated to COG- and Pfam-based functions, respectively. For an overview of predicted COG categories see Table 4.

**Table 3:** Genome statistics

Attribute	Value	%
Genome size (bp) ^a^	3,088,407	100
DNA coding (bp)	2,621,999	84.9
DNA G + C (bp)	1,164,329	37.7
DNA scaffolds	1281	100
Total genes	3097	100
Protein-coding genes	3045	98.3
RNA genes	46	1.5
Pseudo genes	6	0.2
Genes in internal clusters	-	-
Genes with function prediction ^b^	2051	67.4
Genes assigned to COGs	1659	54.5
Genes with Pfam domains	1984	65.2
Genes with signal peptides ^c^	337	11.1
Genes with transmembrane helices	626	20.6
CRISPR repeats	10	

^a^All 1281 scaffolds >200 bp. 478 of these (37.3%) are scaffolds >1000 bp, comprising 2,726,561 bp (88.3% of all base pairs)

^b^Genes with function prediction are all 3045 protein-coding genes minus those 994 genes annotated as “hypothetical proteins” that have no COG category or fall into the COG categories “unknown function” or “general function prediction only” and that have no Pfam domain or a Pfam “domain of unknown function”

^c^Includes genes for which a signal peptide was predicted with at least two of the three tools used. Percentages of genes with function prediction, COGs, Pfam domains, signal peptides and transmembrane helices were calculated against a total of 3045 protein-coding genes

**Table 4:** Number of genes associated with general COG functional categories

Code	Value	% age	Description
J	179	5.88	Translation, ribosomal structure and biogenesis
A	1	0.03	RNA processing and modification
K	50	1.64	Transcription
L	126	4.14	Replication, recombination and repair
B	0	0.00	Chromatin structure and dynamics
D	20	0.66	Cell cycle control, cell division, chromosome partitioning
V	81	2.66	Defense mechanisms
T	40	1.31	Signal transduction mechanisms
M	105	3.45	Cell wall/membrane biogenesis
N	6	0.20	Cell motility
U	47	1.54	Intracellular trafficking and secretion
O	99	3.25	Posttranslational modification, protein turnover, chaperones
C	115	3.78	Energy production and conversion
G	34	1.12	Carbohydrate transport and metabolism
E	117	3.84	Amino acid transport and metabolism
F	46	1.51	Nucleotide transport and metabolism
H	104	3.42	Coenzyme transport and metabolism
I	46	1.51	Lipid transport and metabolism
P	61	2.00	Inorganic ion transport and metabolism
Q	98	3.22	Secondary metabolites biosynthesis, transport and catabolism
R	175	5.75	General function prediction only
X	32	1.05	Mobilome: prophages, transposons
W	9	0.30	Extracellular structures
S	68	2.23	Function unknown
-	1386	45.52	Not in COGs

The percentage is based on a total of 3045 protein-coding genes

## Insights from the genome sequence

Sulfur-oxidizing symbionts of *Bathymodiolus* species are assumed to be horizontally transmitted, i.e., they supposedly enter their bivalve hosts from a free-living bacterial population in the environment, rather than being transferred from one mussel generation to the next [39]. The idea of a putative free-living stage of the symbiont in the hydrothermal vent environment is in accordance with our genome analysis: Unlike some insect symbionts, which are obligatorily dependent on their hosts and have a diminished genome [40], the *B. thermophilus* symbiont genome (3.1 Mb in size, see below) is not reduced. With the exception of the tricarboxylic acid cycle, which lacks three enzymes (see below), all necessary pathways for a host-independent life-style appear to be complete in the *B. thermophilus* symbiont’s genome.

### Energy generation

The *B. thermophilus* symbiont uses reduced sulfur compounds such as sulfide and thiosulfate as its major energy sources [10]. As predicted from the genome sequence, sulfide and thiosulfate are oxidized to sulfate via the rDSR-APS-Sat pathway and the Sox multienzyme-complex, respectively. Oxygen and nitrate are used as final electron acceptors. Complete gene sets for these pathways are present in the symbiont genome.

### CO_2_ fixation and carbon metabolism

The *B. thermophilus* symbiont genome furthermore encodes a modified version of the CO_2_-fixing Calvin-Benson-Bassham cycle: while the genes for sedoheptulose-7-phosphatase and fructose-1,6-bisphosphatase are missing, a pyrophosphate-dependent 6-phosphofructokinase is encoded, which potentially replaces the two other functions (as also described for the endosymbionts of *Calyptogena magnifica* [12], *Riftia pachyptila* [13] and *Olavius algarvensis* [16]). The *B. thermophilus* symbiont’s TCA cycle is incomplete, as the enzyme 2-oxoglutarate dehydrogenase is missing. Moreover, homologs of the enzymes malate dehydrogenase and succinate dehydrogenase are also lacking, similar to what was reported for the thiotrophic *B. azoricus* symbiont [17].

### Nitrogen metabolism

The *B. thermophilus* symbiont possesses genes for assimilatory nitrate reduction, i.e. for nitrogen uptake from nitrate. Its genome also encodes the Nar complex, a membrane-bound respiratory nitrate reductase necessary for respiratory reduction of nitrate, indicating that nitrate can be used as an alternative electron acceptor besides oxygen. Several membrane transporters for the uptake of nitrate, nitrite and ammonia are also encoded.

### Immunity and cell surface interactions

Of the 3045 protein-coding genes, 10.74% are predicted to contain Pfam domains related to bacterial cell surface adhesion, such as bacterial Ig-like domain proteins and cadherins, and to putative toxins, such as pore-forming RTX and MARTX cytotoxins. Another 2.17% of the protein-coding genes were associated with immunity against phages (CRISPR-Cas, restriction modification system and the Abi toxin-antitoxin system). This elaborate presence of genes associated with pathogenicity and phage defense, typical of pathogens and bacteriophages, was also observed in the related thiotrophic *B. azoricus* symbiont [17, 41]. This particular feature of *Bathymodiolus* symbionts is surprising since the bacteria a) reside in shielded intracellular niches, b) are beneficial symbionts for their host, and c) are not related to any known pathogen [26, 41]. Moreover, approximately 1.71% of the protein-coding *B. thermophilus* genes belonged to several classes of pathogenic and digestive peptidases. Membrane transporters of type I and type II secretion systems, which transport toxins and folded exoproteins such as peptidases, are also encoded. Although their exact roles have not been determined as yet, we postulate that these pathogenicity-related genes may be involved in protecting the symbionts against pathogens or phages or even perform symbiosis-specific functions, such as symbiont attachment to the host or defense against the host’s immune system, as suggested previously [41].

## Conclusions

Sequencing of the uncultured *B. thermophilus* symbiont’s genome allowed preliminary insights into its genomic characteristics and metabolic potential. *Candidatus* Thioglobus thermophilus appears to solely rely on sulfide and thiosulfate as energy sources, as genes for the oxidation of other reduced compounds were absent from its genome. The absence of three genes encoding essential TCA cycle enzymes, which was recently also reported for the thiotrophic *B. azoricus* symbiont [17], may suggest that these genes are consistently missing in *Bathymodiolus* symbionts. The unusual presence of a repertoire of genes associated with cell adhesion, toxin production and phage immunity in the non-pathogenic *B. thermophilus* symbiont may point to a symbiosis-specific beneficial role of these functions other than pathogen defense.

## Abbreviations

APS: Adenylyl-sulfate reductase
EPR: East Pacific Rise
rDSR: Reverse dissimilatory sulfite reductase
Sat: ATP sulfurylase
Sox: Sulfur oxidation
TCA cycle: Tricarboxylic acid cycle
